# Don’t assume, ask! A focus group study on end-of-life care planning with people with intellectual disabilities from minoritised ethnic groups

**DOI:** 10.1186/s12904-025-01646-0

**Published:** 2025-01-14

**Authors:** Andrea Bruun, Leon Jordan, Jo Giles, Rhidian Hughes, Rebecca Anderson-Kittow, Irene Tuffrey-Wijne

**Affiliations:** 1 Kingston University London, London, United Kingdom; 2 Voluntary Organisations Disability Group, London, United Kingdom; 3 Kenry House, Kingston Hill Campus, Kingston upon Thames, London, KT2 7LB United Kingdom

**Keywords:** End-of-life care planning, Palliative care, Intellectual disability, Death, Social care, Social services, Minoritised ethnic groups

## Abstract

**Background:**

People with intellectual disabilities are less likely to have access to palliative care, and the evidence shows that their deaths are often unanticipated, unplanned for, and poorly managed. Within the general population, people from minoritised ethnic groups are under-represented within palliative care services. End-of-life care planning with people with intellectual disabilities from minoritised ethnic groups may be a way to address these issues. There is a huge gap in the evidence regarding intersectionality of intellectual disability and ethnicity within end-of-life care planning. This study explored the characteristics of effective and preferred end-of-life care planning approaches and resources for people with intellectual disabilities from minoritised ethnic groups.

**Methods:**

Nine focus groups and three semi-structured individual interviews were held with 41 participants from minoritised ethnic groups (11 family carers; 25 support staff; and five people with intellectual disabilities). Session recordings were transcribed verbatim and analysed using the framework analytical approach.

**Results:**

Participants thought that end-of-life care planning practices were dependent on the person’s culture, ethnicity, and religion, and that it was important to follow these at the end-of-life. They deemed it important to discover and respect (and not assume) the individual’s perspectives, values, needs, and wishes through a person-centred approach. Cultural attitudes to talking about death could hinder end-of-life care planning as participants perceived it as taboo. Disagreement was described as hindering end-of-life care planning, particularly when strong feelings about cultural and religious practices were involved. Staff highlighted the need for cultural and religious awareness, which could involve seeking information and receiving training. Opening the conversation about death and dying was seen as a potential facilitator for exploring end-of-life care planning.

**Conclusions:**

The study was committed to addressing issues of equity, diversity, and inclusion. It is the first study to explore perspectives on end-of-life care planning with people with intellectual disabilities from minoritised ethnic groups. It was deemed important that staff did not assume but discovered and respected the views and preferences of people with intellectual disabilities regarding culture and religion. There is an urgent need for more research into end-of-life care planning with people with intellectual disabilities from minoritised ethnic groups.

**Supplementary Information:**

The online version contains supplementary material available at 10.1186/s12904-025-01646-0.

## Background

Providing a culturally sensitive service is crucial in ensuring appropriate end-of-life care services, where it is particularly important to ensure that services are sensitive to and consider the cultural needs of the person with intellectual disabilities [1]. As approaches to end-of-life care and planning are potentially culturally sensitive and diverse, it is necessary to explore the preferences and perspectives on end-of-life care planning from people with intellectual disabilities from minoritised ethnic groups.

Addressing the inequalities in accessing palliative care for people with intellectual disabilities has been labelled an urgent priority globally [2]. National mortality reviews and studies from the United Kingdom have shown that the deaths of people with intellectual disabilities are often unanticipated [3, 4], unplanned for, and poorly managed [5]. Recent reports found that people with intellectual disabilities from minoritised ethnic groups die at a younger age in comparison to those of white ethnicity [6–8]. As a response, NHS England has prioritised work that promotes understanding of the challenges faced by people in accessing services and establishing links with minoritised ethnic groups, to learn how to address additional inequalities relating to race and ethnicity [9].

Within the general population, patients and families from minoritised ethnic groups are under-represented within palliative care services internationally [10]. Potential explanatory factors for the low uptake of palliative care services include lack of referrals, lack of knowledge about services, and religious traditions and family values that may potentially conflict with the principles of palliative care [11]. Language barriers and a lack of cultural understanding have also been highlighted [12]. People from minoritised ethnic groups who also have an intellectual disability may experience inequalities based on both factors [7]. Thus, the intersectionality of intellectual disability and ethnicity within palliative care warrants urgent and significant investigation.

End-of-life care planning may be a way of preventing these poorly managed deaths, and reviews and inquiries consistently recommend that services plan for end-of-life care, involving people with intellectual disabilities and families [13, 14]. However, research into end-of-life care planning with people with intellectual disabilities has not focused on those from minoritised ethnic groups [15], where the body of research either involves participants mainly of white (European) ethnicity [16, 17]or ethnicity of participants is not reported [18, 19]. There is a prominent and urgent need for addressing this research gap. For this reason, the aim of this study was to explore and understand the characteristics of effective and preferred end-of-life care planning approaches and resources for people with intellectual disabilities from minoritised ethnic groups.

## Methods

A focus group study with a phenomenological research design, with content analysis using the framework method [20] was conducted to address the study aim. The Consolidated Criteria for Reporting Qualitative Research reporting guideline was followed [21].

This study was part of a wider project to develop an end-of-life care planning toolkit with people with intellectual disabilities, which included a focus group study [16]. As part of the project development process, and as the initial sample was insufficiently diverse, additional funds were made available to conduct further focus groups with an Equity, Diversity, and Inclusion (EDI) perspective.

### Research team

The research team consisted of a Professor [IT-W] with 20 + years’ experience in end-of-life research involving people with intellectual disabilities; two Research Associates [AB, RA-K] with experience in co-producing qualitative research within palliative care; a Research Assistant with intellectual disabilities [LJ] [22] with some research training [23] and 1.5 years of end-of-life research experience; a Research Assistant [JG], with end-of-life experience, supporting [LJ]; and the Chief Executive of a national membership organisation that brings together third sector/not for profit disability service providers, who is a former social policy academic who has also worked in the field of palliative care research [RH]. This team was part of a wider research team including three more researchers with intellectual disabilities and intellectual disability service managers.

This study was carried out in close collaboration with the Voluntary Organisations Disability Group (VODG), a national membership organisation that brings together third sector/not for profit disability service providers. In collaboration with VODG, an Advisory Board was formed to inform and guide the study. The board included people with intellectual disabilities, family members, and support staff from minoritised ethnic groups. The board met twice; at the beginning of the study and following completion of data collection and analysis where they verified study findings. There are a wide range of views and definitions on issues of equity, diversity, and inclusion. During the first meeting, the Advisory Board agreed on using the term “minoritised ethnic groups”, which was adopted throughout this study.

To mitigate the position of the lead researcher [AB] as an individual with a European white background conducting a study exploring the views of minoritised ethnic groups, several actions were taken alongside the establishment of the Advisory Group. EDI experts both within the host university and outside the university were contacted to advise on how to conduct the study in a sensitive and appropriate way. Relevant resources and guidelines were followed [24, 25]. Having focus group co-facilitators from minoritised ethnic backgrounds were put in place. AB met up with the co-facilitators prior to the focus groups to go through the study materials and procedures.

### Participants and recruitment

Participants were purposively selected to take part in single-stakeholder focus groups, including adults with intellectual disabilities, families, and intellectual disability support staff.

Participants had to be adults, and either have intellectual disabilities themselves, be a family member of someone with an intellectual disability or be social care support staff supporting someone with intellectual disabilities. In the United Kingdom, adult social care are broad services that enable people with intellectual disabilities to maintain their independence and well-being, and also include the provision of regulated personal care.

All participants had to be from a minoritised ethnic background or be supporting someone with intellectual disabilities from a minoritised ethnic group to be eligble for the study. Participants also had to understand and speak English to participate in the study. No prior knowledge or experience of end-of-life care planning was needed.

VODG helped establish networks with relevant organisations that work with people with intellectual disabilities from minoritised ethnic groups. Members of the wider research team also used their network connections to find eligible study participants. Relevant stakeholders were contacted via email to see if they had an interest in participating. A recruitment flyer and Participant Information Sheet were developed. Easy-read and video study materials were developed for the focus group with people with intellectual disabilities. Organisational Managers passed on study information, and informed consent was then obtained by the research team.

A “People First” group for people with intellectual disabilities agreed to participate in the study. All but one member came from a minoritised ethnic background. To avoid excluding that member from the group, as the member was a key group member (and founder), it was agreed that the person could participate despite not being from an ethnic minoritised group.

### Ethical considerations

Approval for the study was obtained from West Midlands – Coventry & Warwickshire Research Ethics Committee (22/WM0026) on 22/04/2022. Participants had to provide written consent to participate in the study.

People with intellectual disabilities had support to go through the study information materials to ensure they understood the study and what their involvement entailed.

### Data collection

Focus groups were held either in person (*n* = 4) or online on Zoom (*n* = 5). In-person focus groups with intellectual disability support staff were held in one of the organisation’s meeting rooms. The focus group with people with intellectual disabilities lasted four hours, while the other groups lasted two hours. The research team decided to offer the option of conducting individual interviews with participants to accommodate the difficulties and delay with recruiting participants and staff changes within the research team. Individual interviews were all held online on Zoom (*n* = 3) and lasted approximately one hour.

The focus group with people with intellectual disabilities was conducted using some of the inclusive research methodologies developed for the wider focus group study [16] (including ice-breaker games). Thus, more time was needed for this group. A new and creative approach was developed with the research team, including the researcher with intellectual disabilities, which involved inviting participants to draw a funeral. Participants were also shown funeral pictures, which had been developed for the end-of-life care planning toolkit as part of the wider research project [16]. Comments were invited to understand whether the pictures were workable within a more diverse population and could elicit people’s different perspectives. The focus group with people with intellectual disabilities was co-facilitated with the researcher with intellectual disabilities from a minoritised ethnic background (LJ with support from JG). The group was held in the group’s local community office. Two non-research participant Support Workers were present to support the participants.

Focus groups and interviews with family carers and intellectual disability staff were conducted using a topic guide eliciting participants’ perspectives on end-of-life care planning. The topic guides were developed for the wider focus group study and separate ones were created for this study to include specific questions about ethnicity, culture, and religion and to inform the current stage of the wider project. The topic guides can be found in Additional File 1. Focus groups and interviews were facilitated by one researcher [AB]. Two focus groups with support staff were co-facilitated by a Support Worker from a minoritised ethnic background working for the particular organisation. The co-facilitator was not available to facilitate the third focus group held within the organisation. Due to low numbers of participants, it was agreed that having a co-facilitator would seem overwhelming and therefore not appropriate for the remaining focus groups held in two other organisations.

All focus groups and interviews were recorded (audio or video). Researchers wrote field notes during and/or after each session. Data were collected from April-June 2023.

### Data analysis

Recordings were professionally transcribed verbatim using a secure transcription provider. Data were analysed using content analysis, following the framework method which has the advantage of being adaptable and allowing engagement of people without qualitative research experience [20]. The analytic progress included group analysis days with the research team, including the researcher with intellectual disabilities.

A matrix of deductive codes was created based on the topic guides (see Additional File 2). Following each focus group/interview, AB populated the sections. Inductive codes were added to the matrix. After the final data entries, transcripts were re-read to check that all themes were represented in the analysis. The findings were discussed with and verified by members of the Advisory Board.

## Results

A total of nine focus groups and three individual interviews were held with 41 participants (two focus groups and two individual interviews with 11 family carers; 6 focus groups and one individual interview with 25 support staff; and one focus group with five people with intellectual disabilities). Participants’ demographics are displayed in Table 1.

**Table 1:** Participants’ demographics

Demographic information	Total	People with intellectual disabilities	Support Workers^1^	Family members
N (%)	41 (100)	5 (10)	25 (61)	11^2^ (27)
Age (years)				
20–29	2 (5)			2 (18)
30–39	4 (10)		4 (16)	
40–49	9 (22)	2 (40)	7 (28)	
50–59	6 (15)	1 (20)	5 (20)	
59>	12 (29)	2 (40)	1 (4)	9 (82)
Not provided	8 (20)		8 (32)	
Gender				
Female	27 (66)	2 (40)	19 (76)	6 (55)
Male	13 (46)	3 (60)	5 (20)	5 (45)
Do not want to say	1 (2)		1 (4)	
Nationality				
British	27 (66)	5 (100)	12 (48)	10 (91)
Dutch	1 (2)		1 (4)	
Ethiopian	1 (2)		1 (4)	
Jamaican	1 (2)		1 (4)	
Macedonian	1 (2)		1 (4)	
Nigerian	2 (5)		2 (8)	
Polish	1 (2)		1 (4)	
Romanian	1 (2)		1 (4)	
Dual nationality^3^	2 (5)		2 (8)	
Not provided	4 (10)		3 (12)	1 (9)
Ethnicity				
White/White British^4^	14 (34)	1 (20)	6 (24)	7 (64)
Asian/Asian British	3 (7)	2 (40)		1 (9)
Black/Black British	14 (34)	1 (20)	12 (48)	1 (9)
Mixed background^5^	3 (7)	1 (20)	2 (8)	
Other ethnic background^6^	6 (15)		4 (16)	2 (18)
Do not want to say	1 (2)		1 (4)	
Experience working within the intellectual disability sector (years) (*n* = 25)				
≤ 10	11 (44)		11 (44)	
11–20	9 (36)		9 (36)	
20>	3 (12)		3 (12)	
Not applicable	1 (4)		1 (4)	
Not provided	1 (4)		1 (4)	

^1^ Included the following roles “Support Worker”, “Community Connections Support Worker and Care Worker”, “Community Connections Support Worker”, “Operational Manager”, “Service Manager”, “Registered Home Manager”, “Home Manager”, “Operations Manager”, and “Group Work Coordinator”

^2^ Family members were either parents (*n* = 8) or siblings (*n* = 3) of a person with intellectual disabilities

^3^ Included “British/African” and “British/Nigerian”

^4^ Included “White/White British – Polish”

^5^ Included “Asian/White British - (British/Ghanian)” and “White/Black Caribbean”

^6^ Included “Jewish”, “White/Other”, “Black African”, “White/East European”, and “Filipino”

### Funeral planning is culturally dependent

Participants mentioned funeral planning in relation to end-of-life care planning. They expressed how they viewed funeral planning as being dependent on both culture and religion, with certain practices, traditions, and different ways of doing things.

*I am from Jamaica*,* I’m West Indian. So my way of looking at funerals might be different from somebody who comes from*,* say Romania or a Jewish person. You know there might be similarities*,* but they’re not exactly the same.*

*(Support Worker and Service Manager*,* SE601)*

Participants talked about their end-of-life care planning experiences related to culture, ethnicity, and religion. These included being members of the black community (participants used the term “black funerals”); being Cypriot, Jamaican, Polish, and Romanian; and being Catholic, Christian, Jewish, and Muslim. One person with an intellectual disability explained.

*Because if you are a different religion*,* some religion*,* they wash them up*,* but I don’t know about the Christian*,* how they do them. But my religion is Muslim*,* because when we die*,* then we wash them up*,* and then after that we wrap them up in a material*,* and then after that we put them into the coffin box*,* and then we put the special spray in.*

*(Person with intellectual disabilities*,* LE104)*

This also involved different degrees of planning. People explained how they had not done any planning and did not intend to (in some cultures this was left to the family). Some people said that family members in their country would have bought coffins and have them ready in the attic. Others explained how they had pre-paid, fully arranged funeral plans in place from an early age.

*In my own experience back home*,* our people don’t make end-of-life planning beforehand. No*,* they only write wills giving out material property to family members. After you have died it’s only the family members who have to organise about your funeral and how it’s going to be. Yes*,* it’s not in plan. They don’t plan it beforehand.*

*(Support Worker*,* SE4x)*

*I have signed up for the burial system scheme from the United Synagogue. So that’s a scheme offered to Jewish people to pay for their funeral. It’s a very practical scheme to pay for their funeral in advance. So we’ve paid the money*,* and the idea about that is attached to my will and the people that know where my will is*,* assuming that I’ll go first*,* will find that. And from a practical point of view his funeral has been sorted out.*

*(Mother*,* FE802)*

It was deemed important that a person’s culture and religion were followed and respected at the end-of-life.

*I think it’s important to include all those factors - religion and culture - because*,* obviously*,* that’s a person’s identity. As a Support Worker*,* I think it’s a part of our job to make sure we are supporting service users to have a dignified funeral service and that all of those aspects are included in making the right decision for them.*

*(Support Worker*,* SE502)*

### How to do end-of-life care planning

#### Respecting the individual’s practices and wishes

Building upon the theme of the importance of culture and religion at the end-of-life, participants deemed it important when doing end-of-life care planning that the individual’s cultural and religious practices and wishes were respected. The individual’s wishes may differ from more well-known practices of the culture, or Support Workers’ own views, and they expressed how it was important to respect the person’s preferences.

*I recognise that everybody’s different*,* everybody’s got different ways of doing things. I might not agree with it*,* but they do agree with it*,* and as long as it’s safe*,* that’s my main concern. I won’t disagree*,* or disrespect*,* or argue against another person’s opinion or choice.*

*(Support Worker*,* SE304)*

In some cultures, going against someone’s wishes was seen as a very bad thing.

*In my culture*,* people that are very fanatic with belief*,* they will say*,* ‘This person said…’ let’s say a woman died*,* a wife died*,* and the husband say*,* ‘You must bury me beside my wife when I die.’ So if the husband says bury beside the wife*,* and the family say no*,* they don’t want to grieve at the same place. ‘I don’t want to bury this man there.’ We have to - if you go against the person’s wish*,* in my culture*,* something bad happens. I don’t know how it happen*,* but they say when something happens*,* they will now point a finger and say*,* ‘This is because you didn’t respect his wish. He wanted his grave to be beside the wife.’ So that is to tell you how powerful respecting people’s individual wishes is.*

*(Support Worker*,* SE4x)*

#### Person-centred approach

Participants explained how culture, ethnicity, and religion were individualised, and therefore they believed an individual person-centred approach should be taken. Despite certain traditions and practices, it was deemed important that the person with intellectual disabilities was at the centre, and it was about what *they* would want; sometimes maybe contradicting with cultural and religious norms and rules.

*Religious Jewish people would have a different view from lesser religious Jewish people. So it’s not something that would be important to me*,* but it might be to others*,* and that’s something that I think needs looking at.*

*(Mother*,* FE802)*

They explained how it was important not to make assumptions about the person and their wishes.

*Again*,* cultural differences - and when I say cultural*,* I’m not just talking about that from a black man. It may be that they’ve lived in the west side of the country*,* or up north in Scotland. There are differences*,* so we shouldn’t ever make the assumption based on us. We should be quite empathic*,* see things from other people’s point of reference*,* because it may look good to us*,* but it may not be what they want.*

*(Support Worker and Manager*,* SE303)*

As part of the person-centred approach, participants emphasised that the person with intellectual disabilities should be asked about their cultural and religious preferences.

*Obviously*,* the first question is*,* what do you want? What would you want? And then if they started speaking about things like nine nights and oh like yeah*,* like I don’t know*,* I want a still band or I want. Then yeah*,* then you start thinking that it’s about culture*,* or saying to people like in your culture*,* what is it? What is a funeral. What do you guys see as like end of life? And what is it?*

*(Support worker*,* SE1003)*

*I think it’s normalising staff are asking the question*,* what is your culture? I feel like staff just kind of go*,* ‘Oh*,* I don’t want to ask that because it might seem racist or it might seem rude.’ Actually*,* it’s better when people ask*,* ‘Okay*,* yes*,* what do you want me to do for you in terms of your culture?’ Don’t assume that just because I’m a Hindu I don’t eat beef*,* or just because I’m a Muslim I eat only halal or something like that. If staff just normalise just asking the question.*

*(Sibling*,* FE1001)*

Participants also stressed that it was important to let the person with intellectual disabilities know that there are different ways and options available.

To ensure that the individual’s cultural and religious preferences were followed, it was suggested that a person could list what was *not* important to them. This was relevant if certain cultural and religious practices were of less importance and did not need to be followed.

*I’ve never said this before or thought about it before. But what’s equally important is what’s not important. So that they don’t have to worry about thinking. Oh*,* we need to do this*,* but if I don’t care about it*,* then I don’t care about it. And you know that’s useful to know that they don’t have to worry about the things that are not important. Just because it’s in the religion doesn’t mean it’s necessarily important to me.*

*(Mother*,* FE802)*

Having cultural preferences on medical forms or in some kind of template was suggested as well.

*Yes*,* I think it covers patient needs or patient preference*,* but it doesn’t cover cultural. For me*,* those are two separate things because there’s medical preference and then there’s cultural preference and you have to kind of delineate the two. With medical preference being okay*,* I do want to be resuscitated or I don’t want to be resuscitated. I do want painkillers or something like that. Then the cultural side is a whole different - I want this ritual or I want this prayer said or I want this type of food or something*.

*(Sibling*,* FE1001)*

*Making sure like a template that involves all these kind of points - culture*,* gender*,* ethnicity*,* religious background - all these things that is a person’s identity*,* I think*,* is really important*,* yes.*

*(Support Worker*,* SE502)*

#### Cultural and religious awareness

Support staff were aware of the different cultural and religious preferences. However, they acknowledged that they would not know everything.

*I would have to read a lot because I don’t really know much about the concept of dying or even the funeral in other religions like Muslim or Jewish (…) Even how they bury the bodies is completely different. So I would definitely have to prepare myself much more.*

*(Support worker*,* SE202)*

However, they stressed the importance of exploring different cultural and religious practices and seek additional information if needed.

*So I would have to speak to people of all different cultures and religions and find out exactly how it does work for them.*

*(Support Worker*,* SE1003)*

This could involve reaching out to religious leaders as well.

*So thinking about Jewish people in [organisation name] in particular*,* and where [organisation name] is located*,* there is a fair number of synagogues. And there are Rabbis in all these synagogues*,* and whether there could be a Rabbi associated with the charity to oversee that the cultural side of things and the procedures are carried out effectively*,* because that is beyond the remit of the Support Staff and the organisation in a way. So I think you know your local friendly Rabbi for those sorts of things might well be something that’s a good idea.*

*(Mother*,* FE802)*

It was proposed that training should be put in place to support staff to become more aware of the different cultures and religions.

*In terms of the cultural stuff*,* I think it’s just having appropriate training*,* regular training. In the toolkit*,* just showing maybe different cultures*,* like Islam does this*,* they say the Shahada. Hindus do this and they go to India and do a pilgrimage. Just having that explanation to healthcare staff that every culture has a different way of dealing with death.*

*(Sibling*,* FE1001)*

*Training is needed for every person in the company*,* because some people just bring their culture into that*,* and it doesn’t work like that. Because my culture and your culture is not matching*,* so how is that going to happen*,* to do my planning*,* if you don’t know me better?*

*(Support Worker*,* SE301)*

### Barries to end-of-life care planning

#### Death as a taboo

Talking about death and dying was perceived as difficult and even seen as a cultural and religious taboo for many of the participants, across cultures and religions.

*I’m speaking from a South Asian sort of perspective. There’s just not a lot of discussion on it. I don’t really know the reason why. It’s just always been a problem. I think it’s more your parents and your older generation just don’t want to almost even consider that they’re going to leave their family behind. So they just don’t talk about it*,* because if you don’t talk about it then it doesn’t happen*,* kind of thing. Yes*,* it’s also like a whole religious aspect to it*,* in terms of where you’re going*,* where your soul goes and things like that (…) So I think there’s a whole taboo around mentioning the whole topic*,* because people*,* they’re worried about leaving people behind*,* but then they’re worried for their own soul and like what happens to the body and things like that*.

*(Sibling*,* FE1001)*

*The first time I heard the topic come up*,* I just said*,* ‘Oh*,* this one*,* they’re talking about end of life. Some of us will die leaving here.’ That was my first suggestion*,* honestly.*

*(Support Worker*,* SE501)*

*I believe what everybody is planning about mostly is the funeral arrangement*,* but when the person finally pass*,* that one has to be done by fire*,* by force. So this planning ahead of it*,* I don’t really like it. It’s like calling the death*,* calling death on the person. I don’t like it.*

*(Support Worker*,* SE503)*

#### Disagreement

Another barrier was disagreement between parties involved in end-of-life care planning. If there was disagreement, it could be difficult to know whether it was the person with an intellectual disability’s own decision to follow certain practices or traditions. In a religious environment, people with a more liberal approach to life and religious practices could cause tensions and difficulties.

*I think it’s a given that we know that’s what the process is for the United Synagogues. But should somebody be expressing*,* or we believe that they would want something different. Then that’s when*,* yea*,* that is huge. You would have those meetings to plan that change or that amendment to that plan that somebody would have had from*,* as we were saying*,* from childhood.*

*(Support Worker*,* SE703)*

Support staff found it difficult if their views clashed with family beliefs and preferences.

*Now she had got to the stage of end of life where there was nothing but suffering. You know there’s nothing nice about her end of life. But because of the family’s beliefs*,* they would not agree to a do not resuscitate*,* because you preserve life at all costs. And that’s really hard to in an emotional situation*,* to be told that you know the family are the only ones that can agree to this but in your opinion*,* they are allowing someone to suffer because of their religious beliefs.*

*(Support Worker*,* SE701)*

#### Religious practices

Culture and religion could also be seen as an additional barrier when making end-of-life care plans. Certain cultural and religious practices were seen as very important and as major decisions, especially if the person with intellectual disabilities could not speak for themselves.

*I think without the religious aspect*,* I think it’s almost sort of like you can just take advice from like a funeral company or something*,* about the best sort of action. With the religious aspect*,* it’s almost like - because for example*,* we would go to India and pour the ashes into the Ganges and things like that. I don’t know if my parents would want that*,* if my sister would want that*,* and those are huge commitments to make. So yes*,* I think the religious aspect does complicate it to another level. I mean my mum was very clear*,* just donate my body to science and was like*,* that’s fine. I know my dad would want something more religious or my sister would want something more religious. I can’t directly have that conversation with my sister. So then who do I go to? Do I go to my parents or do I make that decision myself? So there’s a lot of factors to consider.*

*(Sibling*,* FE1001)*

Certain practices could also complicate practical aspects of end-of-life care planning, particularly when these were time sensitive.

*I think with the Jewish religion*,* especially on a Friday*,* it is very hard. Because when they start Sabbath*,* it was very hard to get the Rabbi or anybody to come in.*

*(Support Worker and Service Manager*,* SE604)*

### Facilitators to end-of-life care planning

Helpful ways of doing end-of-life care planning are highlighted in the *how* section (e.g., person-centred approach and cultural awareness).

Participants mentioned opening the conversation about death and dying as a facilitator.

*I think actually that change needs to start with us [younger people] and not with the older gen-*,* because the older generation are just not going to talk about it*,* because they just don’t talk about it. They want that authority*,* and I understand that. I think if we’re going to be the ones to make those final decisions for them*,* then yes*,* we need to almost force them to tell us. Yes*,* we need to demand that they tell us those things.*

*(Sibling*,* FE1001)*

Having these conversations was deemed an important step in breaking the taboo, and participants stressed that it should be done sooner rather than later.

*I think the subject of dying is still a taboo and a hard one for everyone*,* and it would be great to make this subject familiar to everyone. As we speak about our groups of carers*,* I think would be a good idea to introduce it gradually*,* to make it familiar and comfortable to everyone so it’s not a shock*,* it’s not a surprise*,* it’s not emotionally draining. Even now I can feel like my voice is trembling when I remind myself of [name] and her journey at the end. So it is important to*,* I think*,* introduce it quicker and sooner than later.*

*(Support Worker*,* SE404)*

## Discussion

### Summary of study findings

This study showed that participants believed end-of-life care planning is affected by the person’s culture and religion, where significant differences in practices were found between cultures, ethnicities, and religions. Participants thought culture and religion were important at the end-of-life and should be respected and followed. A person-centred approach was proposed, where it was deemed important to discover and respect (and not assume) the individual’s perspectives, values, needs, and wishes alongside their culture and religion. Talking about death was perceived as difficult and a taboo in many cultures, which hindered end-of-life care planning. Disagreement was also found to hinder end-of-life care planning, particularly when strong feelings about cultural and religious practices were involved. Staff highlighted the need for cultural and religious awareness, which may involve them seeking information, getting training, and reaching out to community leaders and other faith-based services. Opening the conversation about death and dying and thereby breaking the taboo was seen as a potential facilitator for doing end-of-life care planning.

### Findings in relation to existing literature

There is an overall gap in knowledge of the impact of ethnicity in the patterns of health and social care service use for people with intellectual disabilities [26]. A limited body of research has explored the intersectionality of intellectual disability and ethnicity and found worse health outcomes and issues with access to services [27, 28]. As this is the first study to explore the perspectives on end-of-life care planning with people with intellectual disabilities from minoritised ethnic groups, there is a need for more research within this area to address the inequalities people with intellectual disabilities from minoritised ethnic groups are facing. More direct and in-depth experiences of end-of-life care planning should be gathered from people with intellectual disabilities, families, intellectual disability staff, health and social care professionals, and community and religious leaders to understand the specific cultural barriers and facilitators. There is a need for interview studies, (retrospective) review of medical files, and observational studies, particularly of how end-of-life care planning conversations happen in practice. Co-developing, -designing, and -delivering research with people with intellectual disabilities and staff from minoritised ethnic backgrounds is essential.

That culture and religion is important at the end-of-life is consistent with other findings from research into the general population [29–31]. It has been shown that for some people it is the religious and cultural preparation for death that is of significance rather than other professional end-of-life care agendas [32].

A guideline into the delivery of high-quality end-of-life care for people with intellectual disabilities recommends not making assumptions, stressing that it is important to assess if the person with intellectual disabilities is a person of faith and whether this informs their expectations about end-of-life care [1]. As every individual’s spiritual needs and wishes are different, it is important to ask about particular spiritual needs related to the end-of-life [33]. This is in line with research stressing that people with intellectual disabilities want to be in control of and make their own decisions about their end-of-life care plans [17], and that they should be supported to enable their involvement in end-of-life decisions [34]. Our study findings confirm this person-centred approach.

National Institute for Health and Care Excellence guidelines for adult end-of-life care for the general population state that a person’s culture and religious beliefs may have a significant influence on whether they wish to be involved in end-of-life care planning discussions [35]. A study showed how some people may consider end-of-life care planning irrelevant and inappropriate because they believed that the timing and manner of their death would be determined by their god [32]. It is recommended that these discussions are approached in a sensitive way, and that it should be respected (and recorded) if someone does not want to engage in such discussions. This is in line with the findings from this study, where it was seen that people plan to different degrees and also that discussing death and end-of-life may be taboo and unwelcomed by people. However, professionals need to remain cognisant that people reaching the end-of-life may wish to return to these topics.

A report into palliative and end-of-life care for people from minoritised ethnic groups recommends that staff training should include cultural competency [11]. The report also recommends open and non-judgemental communication that includes each patient and family being addressed individually and that staff should be aware of stereotypes, differences within the same group, and their own biases [11]. Ten years later this is still the case as a recent study also identified that a lack of cultural and religious literacy and staff’s preconceived stereotypes and “own agenda” were barriers for engaging in end-of-life care planning [32]. Our study, although within an intellectual disability social care setting, had similar results where staff expressed the need for cultural awareness training. This is despite the mandatory Care Certificate in England containing introductory modules on equality and diversity [36], demonstrating that these minimums are not enough to build workforce capability and confidence in addressing end-of-life issues in social care. Specific recommendations to increase people from minoritised ethnic backgrounds in accessing palliative care services have also stressed that information is needed for staff around diet, religion, and the cultural calendar [37].

It has also been recommended to ensure that approaches to end-of-life care include collaboration with local places of worship and faith communities [33]. This aligns with the way of doing end-of-life care planning proposed in this study, where it was suggested to reach out to religious leaders to get more information. Service managers, or the organisation as a whole, should be making strategic links with religious leaders to have support systems in place and share knowledge.

It is worth noting that participants in this study talked mostly about funerals when talking about end-of-life care planning. This is consistent with findings from the wider focus group study on end-of-life care planning, where people with intellectual disabilities, families, and support staff tended to focus on funeral planning rather than planning for care towards the end-of-life [16]. However, this was also seen within a study into end-of-life care planning with ethnically diverse populations without intellectual disabilities [32]. This study similarly described how patients and families oriented to matters to do with funerals and wills (i.e., things that happen after death), rather than what might be anticipated during the process of dying [32]. People seem to better grasp the specific aspects of and explicit concepts of funeral planning than the more abstract and unpredictable aspects of end-of-life illness planning. Future research should further explore the views of people from minoritised ethnic groups on the illness planning part of end-of-life care planning with people with intellectual disabilities.

It should also be considered whether the points presented in this paper are unique to people with intellectual disabilities. This question has also been highlighted in the consensus norms for palliative care of people with intellectual disabilities in Europe [34]. This may suggest that the experiences and views of people with intellectual disabilities from minoritised ethnic groups may not be that different from people without intellectual disabilities from minoritised ethnic groups when looking specifically at culture, ethnicity, and religion and end-of-life care planning.

### Study strengths and limitations

This was the first study to explore end-of-life care planning with people with intellectual disabilities from minoritised ethnic groups. The study involved 41 participants sharing their views and experiences of end-of-life care planning with people with intellectual disabilities from minoritised ethnic groups. The inclusion of different stakeholders (i.e., people with intellectual disabilities, family carers, and Support Workers) was a strength of this study.

The research team experienced difficulties with recruiting participants from minoritised ethnic groups for the study, which resulted in delays. Difficulties may be related to an overall challenge with recruiting people who are willing to discuss end-of-life care planning. Recuritment difficulties were also experienced in the wider study [16]. It is unknown how ethnically diverse care settings are in England, and it may be that low recruitment could also reflect a lack of use of traditionally commissioned social care services by some groups from minoritised ethnic communities. However, there is evidence of systemic racism and ableism in how health, care, and wider welfare is accessed by disabled people in the United Kingdom [38]. Research into this is urgently needed to gain insights into service provision and access for people with intellectual disabilities from minoritised ethnic groups.

It is important to acknowledge that the ethnicity of the researcher facilitating the focus groups may have an impact on the findings. Having a facilitator with similar (minoritised ethnic) background as the study participants could have exposed different perspectives, themes, and insights. It is well-known how there are both strengths and challenges of the researcher being a member of the population they are studying [39]. It is worth noting that our findings align with those from similar studies with people from minoritised ethnic groups without intellectual disabilities outlined in the discussion above. Themes and assumptions were also triangulated through the Advisory Board. Future research should be mindful about focus group facilitators, who the participants are, and the potential impact on the findings. However, there is a need for research to be increasingly co-produced and user-led people from minoritised communities.

Due to a lack of resources, it was not possible to include study participants who did not speak and understand fluent English. This could have excluded potentially interested and otherwise eligible participants who could have provided valuable insights. As it is well-known how language barriers are one of the challenges of providing appropriate end-of-life care for people from minoritised groups, efforts should be made to include them in research. Future studies should mitigate such barriers through translation of study materials and focus groups facilitation.

As participants with intellectual disabilities had to have some verbal ability, people with severe/profound intellectual disabilities could not participate directly in the study. Their views were represented by family members and staff. Future research should further explore the perspectives of people with severe/profound intellectual disabilities from minoritised ethnic groups. Several challenges to obtaining the views of people with severe/profound intellectual disability exist, where ethnographic approaches and comparing the perspectives of the person’s family and professionals have been proposed as possible solutions [40].

## Conclusions

This is the first study to explore end-of-life care planning with people with intellectual disabilities from minoritised ethnic groups. End-of-life care planning was perceived to be culturally dependent, but it is important not to make assumptions about this. To respect and accommodate the individual perspectives, wishes, and wants, a person-centred approach should be taken. It is important that support staff explore the person with an intellectual disability’s preferences. To fully understand the individuals’ preference, support staff should gather information about relevant cultural and religious practices and, crucially, act on these.

## Electronic supplementary material

Below is the link to the electronic supplementary material.

Supplementary Material 1

Supplementary Material 2

## Data Availability

Descriptive metadata will be made available on the Kingston University Research Data Repository in 2025. Full data will not be available, as they are not anonymised. All data requests should be submitted to the Principal Investigator, Prof Irene Tuffrey-Wijne.
