# Supplementary material for: Don’t assume, ask! A focus group study on end-of-life care planning with people with intellectual disabilities from minoritised ethnic groups
Source: BMC Palliat Care. 2025 Jan 14;24:13. doi: 10.1186/s12904-025-01646-0 (PMC11731549; doi:10.1186/s12904-025-01646-0)
Supplement: Supplementary file 1 — Supplementary Material 1 [file 12904_2025_1646_MOESM1_ESM.docx]

# Additional File 1 – Topic guides

**Topic guide: Family carers**

This topic guide will be used flexibly to allow exploration of issues that are important to focus group members and that have been raised in other focus groups.

**Introduction**

Thank you for agreeing to take part in this focus group. We’re going to be talking about end-of-life care planning for family members with intellectual disabilities. Some of this might be difficult to think about and speak about, so if there are any questions you don’t want to answer that’s fine, just let us know. If you want to stop or take a break at any point, that’s fine too [Agree with group on way to signal need for a break].

The reason for running this as a focus group is so that you share similar or different experiences and views with each other. We are really keen to hear from everyone. We are keen to understand how the issues we discuss today are related to people’s ethnic, cultural and faith backgrounds. Please do feel free to talk together about what you think without waiting for us to invite you to speak. It can be tricky online though, so if you can also put your hand up and we’ll make sure you get your chance to speak.

1. Firstly, it would be helpful to know what you think of when we say end-of-life care planning.

*Possible prompts:*

- What do you think this means?
- What topics would/should this cover?

2. Do you think your relative would benefit from end-of-life care planning – now or in the future?

3. What has been your experience (if any) with end-of-life care planning for your family member?

*Possible prompts:*

- Has anyone ever spoken to you or your family member about end-of-life care planning?
- How did you feel about being asked to talk about these things?
- Have you raised the topic with your family member/their support staff?
- What led to this conversation?
- What did you talk about?
- Were you able to discuss your family member’s religious or cultural preferences?
- Were any actions taken as a result of this conversation?
- To what extent do you think this experience was affected by your culture or ethnicity.

3. What would worry you about end-of-life care planning?

*Possible prompts:*

- How do you think your family member would feel about it?
- How would you feel about it?
- Would you feel confident with being involved in this process?
- How do you think those feelings are affected by your, and your family member’s, culture or ethnicity, if at all?

4. When do you think end-of-life care planning should start?

*Possible prompts:*

- Should this only happen when someone is terminally ill, or should everyone have an end-of-life plan?
- If your family member was young and healthy, how would you feel about them having an end-of-life plan?
- How would this process differ for people at different stages of their lives (e.g. young and healthy, older people/those with health issues, and those with a terminal diagnosis)?

5. Who do you think should start conversations about end-of-life planning?

*Possible prompts:*

- e.g. the person themselves, families, staff (if so who), doctors
- Who should be involved in this conversation?
- How much do you think your family member could be involved in the planning process?

5. What do you think is the best way to do end-of-life care planning for your family member?

- How would you/someone start this process?
- How would you/staff involve your family member?
- Are there any materials or resources that would be helpful? (either that you have seen/used or that you think should be made)
- What topics should be covered?
- How could you/staff make sure that your family members’ wishes are understood, recorded and acted on? Including religious and cultural preferences.

6. What do you think helps or hinders end-of-life care planning?

- Do you think your family member’s support staff are open to starting end-of-life care planning? Is there any support or training they would need? Should the training cover culture and ethnicity? If so, what would be important to include?
- Is there any support or training that you feel you need?

**Topic guide: Intellectual disability staff**

This topic guide will be used flexibly to allow exploration of issues that are important to focus group members and that have been raised in other focus groups.

**Introduction**

Thank you for agreeing to take part in this focus group. We’re going to be talking about end-of-life care planning for people with intellectual disabilities. Some of this might be difficult to think about and speak about, so if there are any questions you don’t want to answer that’s fine, just let us know. If you want to stop or take a break at any point, that’s fine too [Agree with group on way to signal need for a break].

The reason for running this as a focus group is so that you share similar or different experiences and views with each other. We are really keen to hear from everyone. We are keen to understand how the issues we discuss today are related to people’s ethnic, cultural and faith backgrounds. Please do feel free to talk together about what you think without waiting for us to invite you to speak. It can be tricky online though, so if you can also put your hand up and we’ll make sure you get your chance to speak.

1. Firstly, it would be helpful to know what you think of when we say end-of-life care planning.

*Possible prompts:*

- What do you think this means?
- What topics would/should this cover?

2. What has been your experience (if any) with end-of-life care planning?

*Possible prompts:*

- Have you (or any of your colleagues) been involved in end-of-life care planning with someone with an intellectual disability?
- What led to this conversation?
- What topics did you/they cover?
- Who was involved?
- Were any actions taken as a result of this process?
- How did you/they feel about this experience?
- What are the requirements in your organisation for end-of-life care planning? Are these considerate of different cultural and religious preferences?
- Have you noticed any differences in the experience of end-of-life care planning with black and minority ethnic people that you or your colleagues support?

3. What would worry you about end-of-life care planning?

*Possible prompts:*

- Would you feel confident with starting this process?
- How do you think people with intellectual disabilities would feel about it?
- How do you think people with intellectual disabilities’ families would feel about it?
- How do you think people’s views of end-of-life care planning are influenced by their culture or ethnicity?

4. When do you think end-of-life care planning should start?

*Possible prompts:*

- Should this only happen when someone is terminally ill, or should everyone have an end-of-life plan?
- How would you feel about starting end-of-life planning with young, healthy people?
- How would this process differ for people at different stages of their lives (e.g. young and healthy, older people/those with health issues, and those with a terminal diagnosis)?

5. Who do you think should start conversations about end-of-life planning?

*Possible prompts:*

- e.g. the person themselves, families, staff (if so who), doctors
- Who should be involved in this conversation?
- How much do you think the people you support could be involved in the planning process?
- What do you think YOUR role is in all this?

5. What do you think is the best way to do end-of-life care planning for people with intellectual disabilities?

- How would you start this process?
- How would you involve people with intellectual disabilities in this process?
- Are there any materials or resources that would be helpful? (either that you have seen/used or that you think should be made)
- What topics should be covered?
- How could you make sure that the wishes of the people you support are understood, recorded and acted on? Including religious and cultural preferences.

6. What do you think helps or hinders end-of-life care planning?

- Do you think staff in your organisation are open to starting end-of-life care planning?
- Do you think families are open to starting end-of-life are planning?
- Is there any support or training you/your colleagues need? How should this include a focus on culture and ethnicity?
