# Supplementary material for: Don’t assume, ask! A focus group study on end-of-life care planning with people with intellectual disabilities from minoritised ethnic groups
Source: BMC Palliat Care. 2025 Jan 14;24:13. doi: 10.1186/s12904-025-01646-0 (PMC11731549; doi:10.1186/s12904-025-01646-0)
Supplement: Supplementary file 2 — Supplementary Material 2 [file 12904_2025_1646_MOESM2_ESM.docx]

# Additional File 2 – Matrix of deductive codes

|  | Focus group 1 | Focus group 2 | Focus group… |
| --- | --- | --- | --- |
| 1. **UNDERSTANDING** |  |  |  |
| 1. **EXPERIENCE** |  |  |  |
| 1. **WHY** |  |  |  |
| 1. **WHEN** |  |  |  |
| 1. **WHO** |  |  |  |
| 1. **HOW** |  |  |  |
| 1. **SKILLS** |  |  |  |
| 1. **BARRIERS** |  |  |  |
| 1. **FACILITATORS** |  |  |  |
| 1. **OTHER** |  |  |  |
| **TEAM THOUGHTS** |  |  |  |
